# Inhibition of host Ogr1 enhances effector CD8^+^ T-cell function by modulating acidic microenvironment

**DOI:** 10.1038/s41417-021-00354-0

**Published:** 2021-06-22

**Authors:** Lin Cao, Weisha Li, Xingjiu Yang, Wenlong Zhang, Mengyuan Li, Haizeng Zhang, Chuan Qin, Xiaohong Chen, Ran Gao

**Affiliations:** 1 Department of Otolaryngology-Head and Neck Surgery, Beijing Tongren Hospital, Capital Medical University, Beijing, China; 2 NHC Key Laboratory of Human Disease Comparative Medicine, Beijing Engineering Research Center for Experimental Animal Models of Human Critical Diseases, Institute of Laboratory Animal Sciences, Chinese Academy of Medical Sciences (CAMS) and Comparative Medicine Center, Peking Union Medical College (PUMC), Beijing, China; 3 National Cancer Center/Cancer Hospital, CAMS & PUMC, Beijing, China

**Keywords:** Tumour immunology, Cancer microenvironment

## Abstract

Immunotherapies for cancer, such as immune checkpoint blockade or adoptive T-cell transfer, can lead to a long-lasting clinical response. But the therapeutic response rate remains low on account of many tumors that have evolved sophisticated strategies to evade immune surveillance. Solid tumors are characterized by the highly acidic microenvironment, which may weaken the effectiveness of antitumor immunity. Here, we explored a promising therapeutic development deployed by pH manipulation for avoiding immunoevasion. The highly acidified microenvironment of melanoma induces the expression of G-protein-coupled receptor (Ogr1) in T cells, which weakened their effective function and promote tumor growth. Ogr1 inhibition reactivate CD8^+^ T cells and have a cytotoxic role by reducing the activity of high glycolysis, resulting in comparatively low acidification of the tumor microenvironment, and leads to tumor suppression. In addition, the adoptive transfer of Ogr1^−/−^-CD8^+^ T cells enhanced the antitumor responses, with the potential for immediate clinical transformation.

## Background

Immune surveillance and effective adaptive immune response are the prerequisites for controlling the malignant transformation of tumor [1]. Surveillance and inhibition of tumors by different types of immune cells have been confirmed, however, the inefficient control of malignant tumors by immune cells is the result of various immunosuppressive mechanisms in the tumor microenvironment (TME) preponderantly [2]. Accordingly, monoclonal antibodies targeting inhibitory signals, such as programmed cell death protein 1 (PD-1) and cytotoxic T-lymphocyte associated protein 4 (CTLA-4) have made remarkable achievements [3, 4]. However, only <30% of patients in the clinic will be helped by these therapies, and it was possible that the TME leads to the maladjustment of tumor-killing activity in immune cells contributes to the growth and development of tumors [5]. For example, abnormal glycolysis of tumor cells can produce excessive H^+^ to render the TME a hostile milieu for T cells [6], which makes it intractable for paralyzed T cells to enter the tumor parenchyma and also leads to tumor regeneration after the immune attack [7, 8]. Under these circumstances, elucidating mechanisms of how to reactivate T cells in acidic TME and play an antitumor role are the core link in tumor immunity.

Proton-sensing G-protein-coupled receptors (GPCRs) have been shown to convert extracellular acids into intracellular signals [9]. Ogr1 (ovarian cancer G-protein-coupled receptor 1), as a member of this family, can sense the extracellular pH (pHe) through histidine residues and fully activated at pHe6.8 [10]. Recent studies have shown that Ogr1 can be expressed on immune cells, and activated Ogr1 leads to functional polarization of tumor-associated-macrophages towards non-inflammatory phenotype, which is responsible for promoting the growth of prostate cancer [10]. Although the role of Ogr1 in cancer has been mentioned, its potential role in regulating the antitumor function of T cells is still unclear.

In this article, we aim to analyze the role of Ogr1 in T-cell-mediated antitumor immunity in the acidic microenvironment. We have found a phenomenon of immune escape arising from G-protein-coupled receptor (Ogr1) negatively regulates cytokines production in an acidic environment, resulting in weakening the effector functions of T cell. In the melanoma model, T-cell-derived Ogr1 but not tumor-intrinsic Ogr1 reduced tumor load and prolonged the survival of mice with lung metastasis. The information gained from this study will also furnish the basis for the development of effective adoptive T-cell therapy for cancer patients by targeting Ogr1.

## Materials and methods

### Mice

The reconstruction of *Ogr1*^*−/−*^ mice is consistent with the method described in the previous literature [11]. C57BL/6 mice were purchased from Beijing Huafukang Bioscience co.inc. *Ogr1*^*−/−*^ and mice carrying LoxP-flanked on both ends of exon1 of Ogr1 locus (Ogr1fl/fl) are from C57BL/6 background, and both of them were obtained from Beijing Weishanglide Biotechnology Co., Ltd. CD4-Cre, CD8-Cre mice, and Rag2^−/−^ mice were all provided by Huafukang Bioscience co.inc. Ogr1^fl/fl^ mice were intercrossed with CD8-Cre or CD4-Cre to obtain Ogr1^fl/fl^CD4^Cre+/−^ or Ogr1^fl/fl^CD8^Cre+/−^ mice. Besides, male mice aged 8–12 weeks were used in vivo and vitro experiments. All mice were bred and maintained in the animal facility of NHC Key Laboratory of Human Disease Comparative Medicine.

### Cell lines

Mouse melanoma cell line (B16-F10) was obtained from the National Infrastructure of Cell Line Resource and cultured with 90% Dulbecco’s modified Eagle’s medium (Gibco, Grand Island, NY) supplemented with 10% heat-inactivated fetal bovine serum (FBS) Gibco, Grand Island, NY) as well as 1% penicillin–streptomycin (HyClone, Logan, Utah, USA). The cells were grown in a cell incubator at 37°C with 5% CO_2_.

To generate stable B16-F10-sh*Ogr1* cell and *Ogr1*-highB16-F10 cells, lentiviruses carrying shRNA against Ogr1 and lentiviral vectors containing full-length mouse Ogr1 cDNA were purchased from Shanghai Genechem Company (Shanghai, China). The RNAi sequence targeting mouse Ogr1 was 5′-GCAGCGTAGCATCAACTACTA-3′. Packaging of viruses was performed and titrated in HEK293T cells according to the manufacturer’s instructions. Lentiviruses containing empty plasmids (vector), Ogr1-overexpressing lentiviruses, and lentiviruses containing nonspecific shRNA were transfected into B16-F10 cells, respectively. All cell lines were not infected by Mycoplasma or other pathogens.

### In vivo tumor experiments

C57BL/6 male mice were injected subcutaneously with 5 × 10^5^ B16-F10 cells (non-targeting gRNA) or 5 × 10^5^ B16-F10-shOgr1cells or Ogr1-highB16-F10 cells, respectively, in 100 µl PBS. *Ogr1*^*−/−*^ mice were injected subcutaneously with 5 × 10^5^ B16-F10 cells (non-targeting gRNA) in 100 µl PBS. When the cells had formed palpable tumors, we monitored tumor growth by caliper measurement with proper blinding every 2 days, and computed tumor volumes as (length × width)^2^/2. Some of the mice were decapitated after 15 days for immunohistochemistry (IHC) analyses. The experiment was terminated when the tumor diameter reached 15 mm. The mice were randomly divided into three groups (*n* = 6 per group).

The tumor-bearing mode of Ogr1^flox/flox^CD4^Cre+/−^ and Ogr1^flox/flox^CD8^Cre+/−^ mice (*n* = 5 per group) was as follows.

*Ogr1*^*−/−*^ and C57BL/6 mice (*n* = 8 per group) were injected intravenously with 4 × 10^5^ B16-F10 cells to construct the model of melanoma lung metastasis and finally were decapitated to assess the number of tumor lesions in lungs after 2 weeks. Randomly selecting one mouse in each group to detect the metastasis by small animal Micro CT (PerkinElmer, IVIS Lumina III) on the twentieth day. Mice were weighed every 3 days and their survival was recorded following tumor challenges.

### Quantitative real-time polymerase chain reaction

The mouse tissues were resected in a biosafety cabinet and then crushed after rapid freezing in liquid nitrogen. Total RNAs were isolated using TRIzol (Invitrogen, CA, USA) and complementary DNAs (cDNAs) obtained using reverse transcription kit and the SYBR Green Master Mix kit (Takara, Otsu, Japan) following the manufacturer’s protocol. The cDNA) as amplified with the following primers: 5′-CTCAATGACCTCCTTGTGATTG-3′ (forward) and 5′-CTACCAGAAAACTCCTCACTATC-3′ (reverse) for TRIM25; 5′-AGGTCGGTGTGAACGGATTTG-3′ (forward) and 5′-TGTAGACCATGTAGTTGAGGTCA-3′ (reverse) for GAPDH; quantitative RT-PCR was carried out an ABI Prism 7900 Sequence detection system (Applied Biosystems, Canada). Relative levels were normalized to that of GAPDH.

### Immunochemical and Immunofluorescence analysis

The fresh tumor was fixed with 10% neutral buffered formalin before paraffin embedding. For histological analysis, 5 mm thick sections were cut and stained with hematoxylin and eosin. Paraffin-embedded sections were stained for NK cells with CD49b (1:100, 108913, BioLegend), B cells with B220 (0.5 µg/ml, ab10558, Abcam), macrophage with F4/80 (1:200, ab16911, Abcam), dendritic cells with CD11c (1:100, ab219799, Abcam), CD4 (1:1000, ab183685, Abcam) and CD8 (1:2000, ab217344, Abcam), as well as LAMP2 (1:500, ab18528, Abcam). A Goat anti Rabbit IgG polyclonal HRP conjugate was used as the secondary. Immunofluorescence staining was applied to assess the presence of tumor-infiltrating CD4 (1:1000, ab183685, Abcam) and CD8 (1:1000, ab217344, Abcam) T cells. Immunohistochemical slices were transformed into high-resolution digital images using Nano Zoomer S60 scanner (Hamamatsu), and image preprocessing and quantitative analysis were performed using NDP.View.2 software. The positive cells of CD49b, B220, F4/80, CD11c, CD4, and CD8 staining were counted after it annotated the tumor region (avoiding necrotic tumor areas). The LAMP2 staining was assessed using a semiquantitative score of 0 (no staining) to 3 (strong staining) by an investigator blinded to the groups.

### T-cell isolation and acidic treatments

Harvesting the spleens of *Ogr1*^*−/−*^ and wild-type (WT) mice and keep them in PBS, and then gently homogenized the spleens with a syringe plunger in a 72 µM sieve. After that, transfer the grinding fluid to a conical tube with centrifugation for 10 mins at 300 × *g*, 4°C. Remove the supernatant and resuspend the splenocytes with red blood cell (RBC) lysis (BD Pharm Lyse, 5075567). The sorting of CD4^+^ T-cell and CD8^+^ T cells Using CD4^+^ T-Cell Isolation Kit and CD8^+^ T-Cell Isolation Kit (Miltenyi, 130–104-454, 130–104–075) following manufacturer’s instructions. Cellular purity was detected with FACSAria II cell flow cytometer (BD Biosciences, CA, USA) after staining with anti-CD3 (1:1000, ab16669, Abcam, Cambridge, MA, USA) and anti-CD4 (1:100, ab133616, Abcam) or anti-CD8a (1:500, ab217344, Abcam) antibody. The data were analyzed with FlowJo softwar. Isolated T cells were cultured in RPM-1640 supplemented with 10% fetal bovine serum (FBS). When adjusting the acidic medium in vitro, we first added 20 mm MOPS (3-(n-morpholinyl) propane sulfonic acid) to the medium containing FBS to minimize the pH change during cell culture and then adjusted the pH value to ph6.6 with 1 N HCl [12, 13]. After 24 hours, the stability of pH value was evaluated by pH meter.

### T-cell proliferation

The sorting of CD4^+^ T cells or CD8^+^ T cells were labeled with 10 μM carboxyfluorescein succinimidyl ester (CFSE) (Thermo CellTrace™ CFSE Cell Proliferation Kit C34554) for incubation at room temperature in the dark for 10 mins. The cells were centrifuged and then washed three times with Roswell Park Memorial Institute 1640 (RPMI 1640) containing 10% FBS. CD4^+^ or CD8^+^ T cells were seeded in 24-well plates (5 × 10^5^ per well) and co-cultured with magnetic beads (Miltenyi, T-cell activation/expansion kit, 130–093–627) of the same proportion in RPMI 1640 supplemented with 10% FBS and IL-2 (50 U/mL) according to the manufacturer’s protocol. After 48 h, 96 h, and 144 h, CD4^+^ T-cell and CD8^+^ T cells were partially harvested and stained with anti-CD4 (1:100, ab133616, Abcam) or anti-CD8a (1:500, ab217344, Abcam). Proliferation was detected by FACSAria II cell flow cytometer (BD Biosciences, CA, USA). The data were analyzed with FlowJo softwar.

### T-cell migration

Transwell assays (costar 3422) were performed to evaluate T-cell migration. B16-F10 cells (5 × 10^5^) were subcutaneously transplanted into C57BL/6 mice and *Ogr1*^*−/−*^ mice. After 15 days, the spleens of each group were harvested to obtain CD4^+^ T cells or CD8^+^ T cells by MACS as a source of migration experiments. CD4^+^ T cells or CD8^+^ T cells (1 ×1 0^6^ per well), suspended in 200 μl RPMI 1640 containing 0.5% FBS, were seeded in the upper chambers. Next, 500 µl RPMI 1640 supplemented with 0.5% FBS and 50 ng/ml SDF (PEPROTECH, 0410173) was added to the lower chambers. T cells were allowed to migrate in a cell incubator for 24 h at 37°C with 5% CO_2_. At last, resuspended the cells in the lower chambers and collected for counting by an automatic cell counter (Life technologies, Countess II, AMQAX1000).

### T-cell-mediated tumor-killing assay

To acquire activated T cells, CD4^+^ T Cells and CD8^+^ T Cells isolated from *Ogr1*^*−/−*^ and C57BL/6 mice were co-cultured with magnetic beads (Miltenyi, T-cell activation/expansion kit, 130–093–627) of the same proportion in RPMI 1640 supplemented with 10% FBS and IL-2 (50 U/mL) for 2 days. B16-F10 cells in 96-well plates (1 × 10^4^ per well) were allowed to stick to the plates overnight and then incubated with activated T cells for 48 h. Different proportions of B16-F10 cells to activated T cells (1:20, 1:40, 1:60) were utilized in this experiment. T cells and their fragments were washed out with PBS, leaving cancer cells for CCK8 experiment (DOJINDO Laboratories, ND657). Then cancer cells were quantified by a spectrometer at OD (560 nm) (BIO-RAD, iMark), stained with crystal violet, and photographed under a microscope (Leica Microsystems CMS GmbH Ernst-Leitz-Str.17–37).

### Cytokine secretion assay

The activated CD4^+^ or CD8^+^ T cells were co-cultured with B16-F10 cells (1 × 10^4^ per well) in 96-well plates at a ratio of 40:1 with the medium of RPMI 1640 supplemented with 10% FBS and 1% penicillin–streptomycin as well as IL-2 (50 U/ml) at 37°C with 5% CO_2_ for 48 h. The content of tumor necrosis factor-α (TNF-α; BD CBA Flex Sets, 558299) interferon-gamma (IFN-γ; BD CBA Flex Sets, 558296) in the cultured supernatant were determined by cytometric bead array (CBA). GramB (R&D systems, DY1865) was analyzed by enzyme-linked immunosorbent assay.

### Adoptive cell transfer therapy

T cells (CD4^+^ T cells or CD8^+^ T cells) for adoptive cell transfer therapies (ACTs) were extracted from the spleens of *Ogr1*^*−/−*^ and WT mice on the 15th day after tumor-bearing (B16-F10, 5 × 10^5^) and stimulated by IL-2 in vitro. Rag2^−/−^ mice were subcutaneous injected B16-F10 cells (2 × 10^5^) and monitored tumor growth by caliper measurement. When the cells had formed palpable tumors, the stimulated T cells (1 × 10^7^) were adoptively transferred into Rag2^−/−^ mice intravenously as a treatment on the seventh and ninth days. Growing melanocytic tumors were measured by caliper measurement every 2 days, and computed tumor volumes as (length × width)^2^/2. The mice were randomly divided into five groups (*n* = 6 per group).

### RNA-seq analysis

CD4^+^T cells and CD8^+^T cells were extracted, respectively, from *Ogr1*^*−/−*^ and WT tumor-bearing mice, and RNA was extracted according to the instructions of Trizol (Invitrogen, Carlsbad, CA, USA). Total RNA was qualified and quantified using a Nano Drop and Agilent 2100 bioanalyzer (Thermo Fisher Scientific, MA, USA). The library was validated on the Agilent Technologies 2100 bioanalyzer for quality control and the final library was amplified with phi29 (Thermo Fisher Scientific, MA, USA) to make DNA nanoball (DNB), which had >300 copies of each molecular. DNBs were loaded into the patterned nanoarray and single end 50 bases reads were generated on BGISEQ500 platform (BGI Shenzhen, China).

### Single-cell RNA sequencing

#### 10x sample processing and cDNA library preparation

To account for interindividual variability about T-cell infiltration, we harvested fresh melanomas from *Ogr1*^−/−^ and WT mice. Tumors were first prepared as a single-cell suspension for 10x genomics processing. After cell suspensions formed droplets, they were transformed into single-cell gel-bead-in-emulsion (GEMs) by 10x Genomics Chromium controller. An RT reaction was carried out in the droplets, followed by demulsification to construct a cDNA library. According to the sample Index, the FASTQ sequence of each sample was first obtained, and then proceed 10x Barcode and UMI sequence as well as insert part (cDNA insert/RNA reads) according to the library structure, finally Barcode was filtered.

### Data processing

The RNA reads section was compared with the reference genome database constructed by STAR alignment software. Based on the alignment of STAR^\[2\]^, we combined it with the information in the reference data set (gtf/gff file). The coverage rate of each region of the genome can be counted, so as to compare the ratio information of exons, introns, and intergenic regions, which can be used as a reference for data quality control.

### Cell clustering

The principal components in the data are calculated. In the factor analysis technique, the variables are grouped according to their correlation, that is, all variables in a particular group are highly correlated, but have low correlation with other groups. Then filter out the most significant principal components according to the degree of enrichment and *p* value. The cells were clustered into several groups by nonlinear dimension reduction analysis based on tSNE or U-MAP (https://www.nature.com/articles/nbt.4314).

### Quantitative statistics of gene expression

The quantitative expression of 10x scRNA-Seq gene was mainly based on UMI counts. If some pairs of reads have the same Barcode sequence and UMI tags as well as alignment to the same position, the PCR repeats will be defined. Only nonPCR repeats, and checked barcodes, as well as UMI, will be used for downstream gene quantitative analysis. The final products were sequenced using the Illumina Hiseq 4000 or Xten platform (BGI_x0002_Shenzhen, China).

### Statistical analysis

Prism 7.0 (GraphPad) was used for statistical analysis to ensure that all statistical tests were reasonable and the differences between the groups were similar. In vivo results were presented as mean ± SEM, and in vitro data were presented as mean ± SD. All in vivo tests were repeated at least twice and in vitro tests in triplicate at least twice. The overall survival rate was analyzed by Kaplan–Meier method, and the differences were analyzed by Log-rank test. Researcher A randomly divided the mice into groups using the random number table method, and Researcher B uniformly manipulated the mice without knowing the grouping. The data are supervised and collated by researchers A and B. No mice were excluded from the analysis as outliers. *P* values <0.05 were considered statistically significant, and statistical significance as *P < 0.05, **P < 0.01, ***P < 0.001.

## Results

### Ogr1 inhibition in host cells mediates tumor suppression

**Fig. 1:**
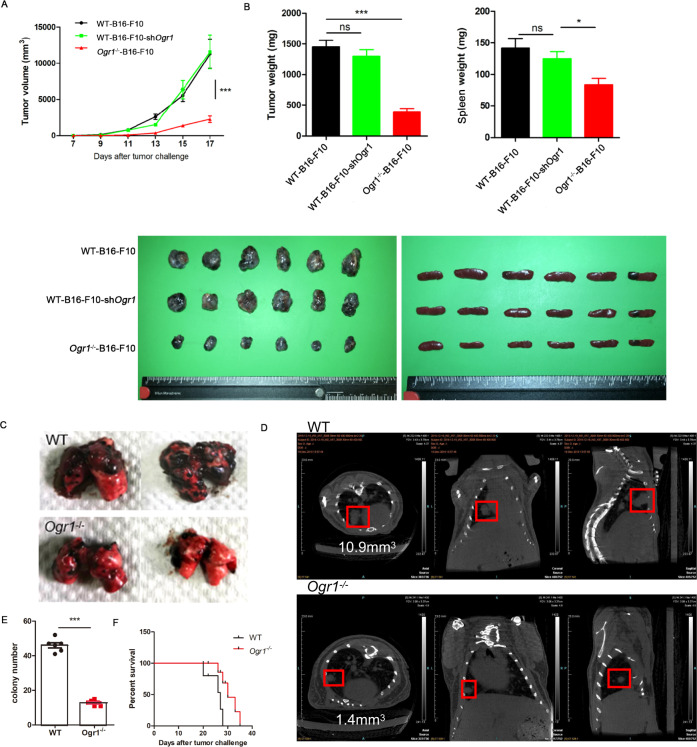
Absence of host Ogr1 specifically strengthens antitumor responses. **A** Tumor growth curves of subcutaneous B16-F10 or B16-F10-sh*Ogr1* in WT(*n* = 6) mice versus *Ogr1*^*−/−*^ (*n* = 6) mice. **B** Weights and volumes of the melanomas and spleens collected on the 19th day. In all, 4 × 10^5^ B16-F10 cells were intravenously injected into WT (*n* = 8) and *Ogr1*^*−/−*^ (*n* = 8) mice. Representative isolated lungs (**C**) and CT images (**D**) as well as the average number of melanoma colonies (**E**) are shown. **F** Kaplan–Meier survival curve that following tumor challenge was recorded. These experiments were repeated three times. The results are presented as means ± SEM. *P < 0.05, **P < 0.01, ***P < 0.001, as obtained by unpaired test.

As a first step to investigate the role of host Ogr1 in tumor development, we established an Ogr1-deficient mouse (*Ogr1*^−/−^) on C57BL/6 background (Supplementary Fig. 1A, B) and used the same background as the WT. Melanoma was chosen as a tumor species because it is particularly suitable for the production of high concentrations of organic acids and H^+^ through glycolysis [14], which leads to strong acidification of the TME [15]. After subcutaneous injection of logarithmic growth of B16-F10 cells to the two groups of mice, tumor growth was evaluated every 2 days for 3 weeks. *Ogr1*^−/−^ mice showed a significant reduction in tumor growth, which reduced the tumor burden by >50% and alleviated tumor splenomegaly (Fig. 1A, B). As Ogr1 can be detected in a variety of tumor tissues in addition to host cells, we also use lentiviral vectors carrying shRNA or *Ogr1*-overexpressing lentiviruses to generate *Ogr1*-knockdown or *Ogr1*-highB16-F10 cells, respectively (Supplementary Fig. 1C, D), to assess the role of Ogr1 in B16-F10 cells in tumor progression. Unlike what we had previously observed in *Ogr1*^−/−^ mice, B16-F10-sh*Ogr1* tumors did not show altered growth compared with WT tumors (Fig. 1A, B) and a high expression of Ogr1 in B16-F10 cells increased the tumor malignancy to some extent (Supplementary Fig. 1E, F) further indicating that Ogr1 expression in host cells may be functionally critical in tumor regression models. In addition, we evaluated the capacity of Ogr1 in the pulmonary metastasis model of melanoma, which indicated that Ogr1 deficiency effectively attenuated tumor metastasis and progression with fewer colonies observed in both anatomical lung tissues and CT images (Fig. 1C–E) (Supplementary Fig. 1G). In addition, >50% of *Ogr1*^*−/−*^ mice had a prolonged survival time (Fig. 1F). Taken together, these data suggest that Ogr1 expression in host cells is responsible for the major, if not all, roles in promoting tumor growth and metastasis in melanoma models.

### Single-cell transcriptomic analysis reveals the link between Ogr1 inhibition in tumor-intrinsic signaling and immunity

**Fig. 2:**
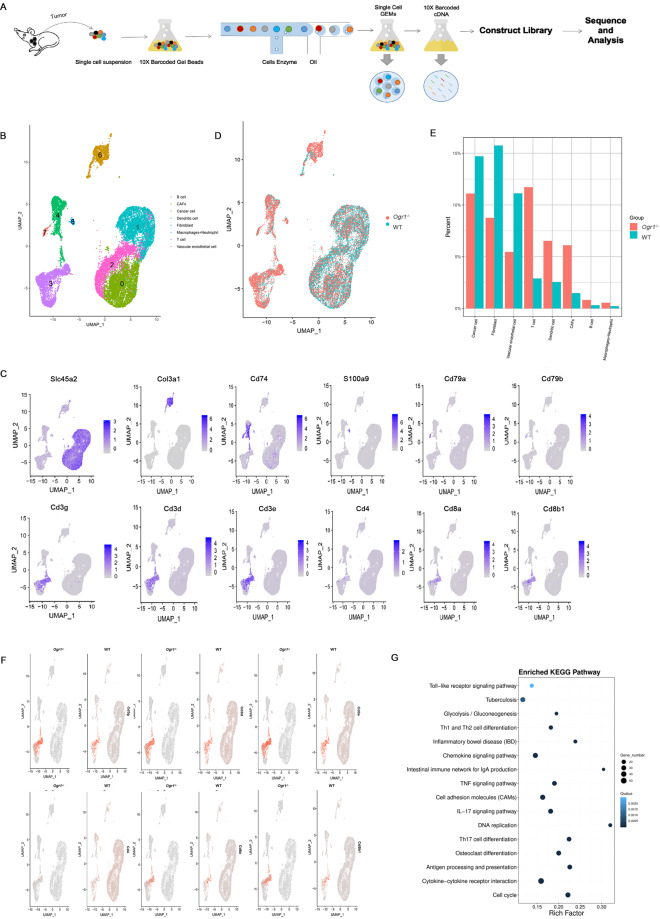
Single-cell sequencing analysis identified intratumoral populations and demonstrated that Ogr1 deletion activate antitumor immunity. **A** Overview of single-cell sequencing workflow. **B** U-MAP displaying confirmed intratumoral populations within two groups merged. **C** U-MAP of representative genes expression in cancer cells and infiltrating cells. **D** U-MAP showed the comparison of cell distribution between the two groups. **E** The percentage of each infiltrating immune population in tumor determined in **B**. **F** U-MAP density map exhibited the distribution of T-cell annotation clusters in the two groups. **G** Enrichment analysis of Kyoto Encyclopedia of Genes and Genomes (KEGG) pathway of differential genes between *Ogr1*^*−/−*^ and WT.

The potential effect of low extracellular pH on immune function reveals the modulation of GPCR pH sensors such as Ogr1 on tumor immune active sites [16–18]. To provide evidence that Ogr1 affects the immune response, we performed single-cell RNA-sequencing (scRNA-seq) analysis of the whole TME including tumor cells and their immune counterparts. We isolated mouse tumors and produced single-cell suspensions in the shortest time. 10×Chromium Controller was used to generate droplets in which cells were lysed and mRNA was collected to form single-cell GEMs. After breaking the emulsions, a cDNA library was constructed, followed by large-scale sequencing. Subsequently, we analyzed single-cell transcriptomes from 9127 cells from the spleens of *Ogr1*^*−/−*^ mice. In all, 11,902 cells from the spleens of WT mice, and 10,279 cells from the tumors of *Ogr1*^*−/−*^ mice, and 8992 cells from the tumors of WT mice. To determine the intratumoral cell type and describe the global immune structural remodeling associated with Ogr1, we computationally analyzed data using the R package Seurat (v 3.1.0) [19]. U-MAP was then used for the two-dimensional visualization of the resulting clusters. For each cluster, the marker genes were identified using the Find Conserved Markers function as implemented in the Seurat package [20] (Fig. 2A, B). According to the Cell Marker database, we distinguished the intratumoral cell and labeled the tumor-relevant cells, including melanoma, tumor endothelial cells, and fibroblasts with *SLC45A2*. Dendritic cells overexpressing the antigen-presenting molecule (MHCII) were labeled with *CD74*, neutrophils, and macrophages with *S100a9*, B cells with *Cd79a* and *CD79b*, and lymphocytes represent T cells with *Cd3* (*Cd3d, Cd3g, Cd3e*) (Fig. 2B, C) (Supplementary Fig. 2A). To distinguish the overall immune landscape remodeling between the two groups, we described the changes in tumor-infiltrating immune cell subsets (Fig. 2D) (Supplementary Fig. 2B). It is worth noting that the frequency of total immune cell components in *Ogr1*^*−/−*^ mice increased compared with that of the WT mice, especially in the number of total T cells and DC cells (Fig. 2E, F). Although neutrophils, macrophages, and B cells occupy only a small segment of the total cell population, this group of cells increased in *Ogr1*^*−/−*^ mice compared with WT cells (Fig. 2E, F). In addition, KEGG pathway analysis showed that *Ogr1*^*−/−*^ had significantly different enrichment pathways compared with the WT group, including antigen processing and presentation, the IL-17-signaling pathway, cytokine–cytokine receptor interactions, Th1 and Th2 cell differentiation, Toll-like receptor signaling pathways (Fig. 2G) (Supplementary Fig. 2C, D). In general, our data emphasize that Ogr1 affects changes in antitumor immune function through the following mechanisms: (1) Ogr1 inhibition triggers a strong immune response signaling, (2) resulting in a significant increase in the number of T-cell infiltrations.

### Ogr1 affects the antitumor immune response mainly through T cells

**Fig. 3:**
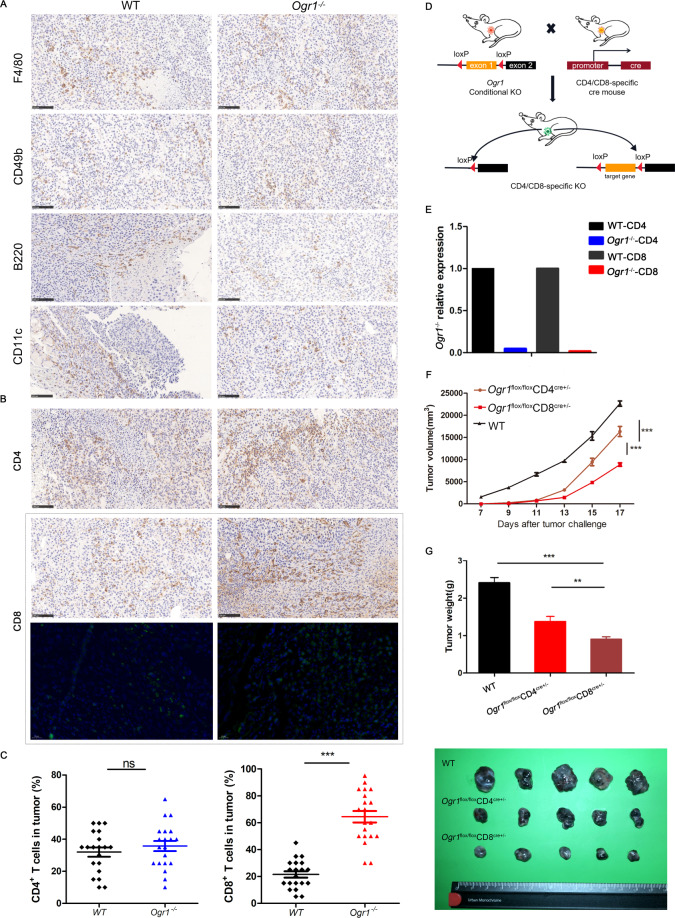
Ogr1 deletion enhances the infiltration of CD8^+^ T cells and contributes to tumor regression. **A** Representative IHC staining was applied to assess the expression of NK cells (CD49b), macrophages (F4/80), B cells (B220) as well as dendritic cells (CD11c) in xenograft melanomas. **B** The representative immunohistochemistry and immunofluorescence staining were applied to assess the number of CD4^+^ T cells and CD8^+^ T cells infiltration in xenograft melanomas. **C** Staining intensities for CD4^+^ T cells and CD8^+^ T cells in melanomas were determined by the assignment of semiquantitative scores. **D** Schematic of mouse models of conditional knockout of *Ogr1* in CD4^+^ and CD8^+^ T cells. When *Ogr1* flox mice mated with CRE mice, the specific exon of *Ogr1* was deleted, thus realizing the conditional knockout of *Ogr1* gene. **E** Ogr1 expression in T cells was detected by RT-PCR. Tumor growth (**F**) and mean tumor volume (Day 20) (**G**) of subcutaneous B16-F10 (5 × 10^5^ in 100 µl PBS) in WT mice versus Ogr1^flox/flox^CD4^Cre+/−^ or Ogr1f^lox/flox^CD8^Cre+/−^ mice. In **A** and **B**, original magnification ×20; scale bar = 100 μm. **F**, **G** experiments were repeated three times. The results are presented as means ± SD. *P < 0.05, **P < 0.01, ***P < 0.001, as obtained by unpaired test.

Single-cell sequencing has shown that Ogr1 affects the function of a variety of immune cells, especially T cells, infiltrating the tumor parenchyma. To verify this, we harvested mouse tumors for immune profiling after 15 days of B16-F10 cell injection. Unfortunately, no significant differences were found in the number of NK cells (CD49b), macrophages (F4/80), B cells (B220^+^), and dendritic cell (CD11c) infiltration between the two groups (Fig. 3A). However, the infiltration of total CD8^+^ T cells in *Ogr1*^*−/−*^ mice was remarkably increased, while no such trend was observed in their total CD4^+^ T cells (Fig. 3B, C). A comparison of the total number of T-cell infiltrations between the two groups revealed that they were consistent with the single-cell sequencing data. To directly correlate Ogr1 expression with the antitumor effect of T cells, we additionally established mouse models with a conditional knockout of *Ogr1* in CD4^+^ and CD8^+^ T cells respectively using the Cre-LoxP system (Fig. 3D), where the correct targeting of the *Ogr1* locus on T cells was confirmed via RT-PCR (Fig. 3E). Tumor growth was evaluated after melanoma cells were implanted into the WT and *Ogr1*^fl/fl^CD4^Cre+/-^ and *Ogr1*^fl/fl^CD8^Cre+/−^ mice, demonstrating considerable tumor suppression after *Ogr1* abrogation from T cells; this suppression was more obvious in *Ogr1*^fl/fl^CD8^Cre+/−^ mice (Fig. 3F, G). These findings further underline that the antitumor immunity produced by Ogr1 inhibition depends on T cells, especially CD8^+^ T cells.

### Ogr1 inhibition attenuates the levels of tumor acidity biomarkers

**Fig. 4:**
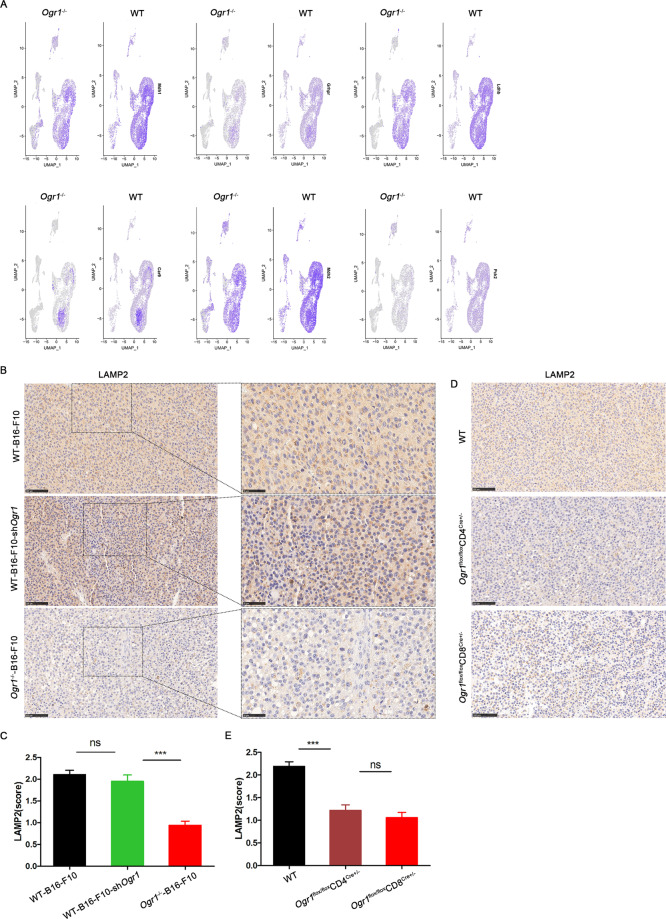
Ogr1 deletion modulates tumor microenvironment acidification. **A** The histogram showed that the expression of genes matching glycolysis activity in single-cell sequencing was compared between *Ogr1*^*−/−*^ and WT. **B**–**E** LAMP2 in melanoma was stained by immunohistochemistry (**B**, **D**) and the staining intensity (**C**, **E**) was evaluated among different groups. Original magnification ×20; Scale bar = 100 μm.

It is known that acidosis in the TME causes cytotoxic T cells to tend to lose their function and remain in a paralysis state followed by apoptosis; therefore, the reversal of T-cell function has become a tricky scientific problem to be solved. Single-cell sequencing analysis showed that the expression of glucose metabolism-related genes in the *Ogr1*^*−/−*^ group was comparatively low, especially in the LDH subunits *Ldhb* and *Pkm2* (Fig. 4A). As an alternative marker of high glycolytic activity, *Ldhb* and *Pkm2* have been proven to be suitable for the production of organic acids and H^+^ [21, 22]. Lysosome-associated membrane protein 2 (LAMP2) [23] has been reported to be positively correlated with TME acidification [24] and regions of high LAMP2 expression in tumors are co-matched with regions of acidosis. To directly correlate the difference in glycolytic activity between *Ogr1*^−/−^ and WT group and the acidification of the TME, we additionally established LAMP2 staining as a substitute for acidic pH. A trend toward lower LAMP2 expression in *Ogr1*^*−/−*^ mice was observed, whereas no difference in LAMP2 expression was observed between B16-F10-shOgr1 and WT mice (Fig. 4B, C). Furthermore, LAMP2 expression in *Ogr1*^fl/fl^CD8^Cre+/−^ mice was also at a low level (Fig. 4D, E), which indicated that the inactivation of Ogr1 in T cells attenuated the acidification of the tumor environment.

### Ogr1 inhibition enhances CD8^+^ T-cell effector functions at acidic pH

**Fig. 5:**
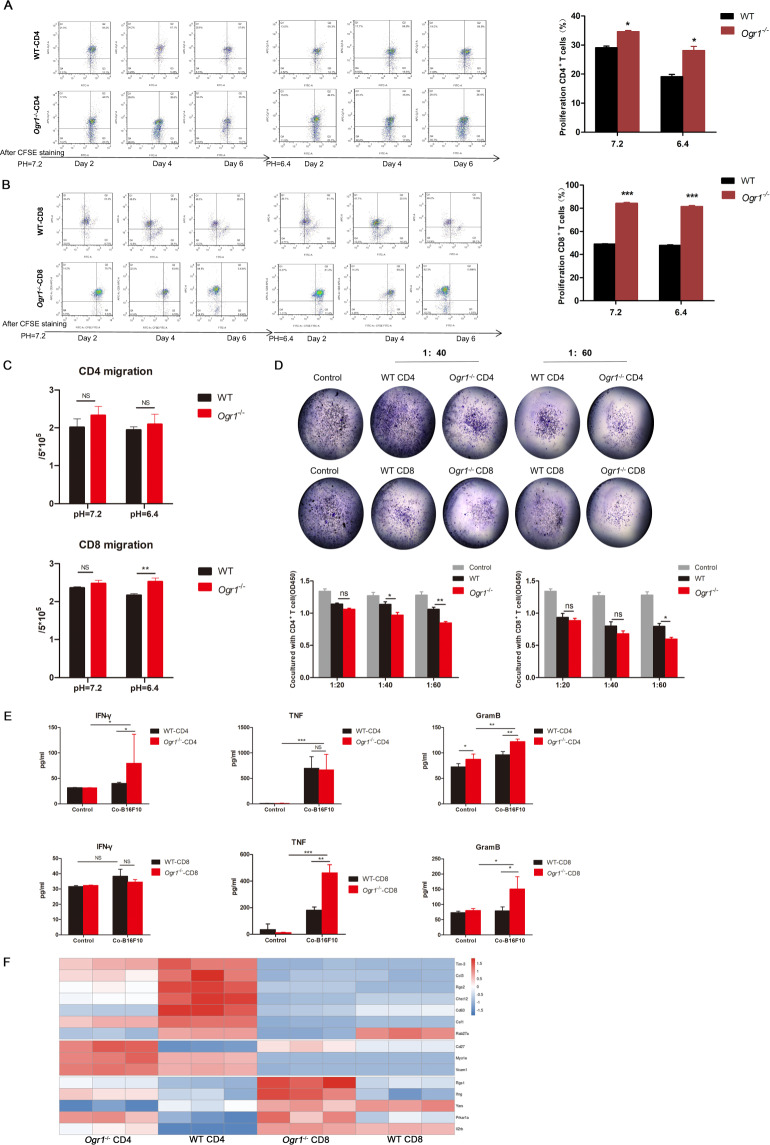
Ogr1 orchestrate the effector function of CD8^+^ T cells. **A**, **B** Representative figures displaying proliferation of CD4^+^ T (**A**) and CD8^+^ T cells (**B**) after anti-CD3 and anti-CD28 antibody stimulation at pH7.2 and pH6.4. **C** Summarized data displaying migration capability of T cells at pH7.2 or pH6.4 by transwell migration assays. **D** Results of T-cell-mediated antitumor effect. B16-F10 co-cultured with activated T-cell for 48 h were conducted to CCK8 followed by crystal violet staining. The ratio of B16-F10 to T cell is 1:20, 1:40, 1:60. A normalized ratio of live cells was shown for each well. **E** The supernatant of co-cultured T cells and B16-F10 cells were collected to detect Granzyme B, TNF-α, and IFN-γ by ELISA. **F** Thermograms included marker genes expression represent T-cell exhaustion and effector/activation in *Ogr1*^*−/−*^-CD4, WT-CD4, *Ogr1*^*−/−*^-CD8, WT-CD8 groups. These experiments were repeated three times. The results are presented as means ± SEM. *p < 0.05, **p < 0.01, ***p < 0.001, as obtained by unpaired test.

To understand the direct consequences of pH alterations on T cells mediated by Ogr1 in regulating tumor protection, T cells isolated from the spleens of B16-F10 tumor-bearing mice (Supplementary Fig. 3A) were cultured for 12 h in the presence of the inorganic acid HCL, which mimics the low pH characteristic of melanomas, with magnetic beads and recombinant IL-2. The gating strategies for fluorescence-activated cell sorting were used to analyze the proliferation of T cells (anti-CD4 or anti-CD8a). The results showed an enhanced T-cell proliferation in *Ogr1*^*−/−*^ mice at pH7.2 (Fig. 5A, B). Although the proliferation of T cells in both groups was inhibited under acidic pH, CD8^+^ T cells (Supplementary Fig. 3B) in *Ogr1*^*−/−*^ mice seemed to render T cells more resistant to low pH than that in WT (Fig. 5B). In addition, transwell assays substantiated that the downregulation of Ogr1 in CD8^+^ T cells noticeably promoted its migration under acidic conditions, whereas no such phenomena were observed in CD4^+^ T cells (Fig. 5C). Next, we conducted an in vitro cytotoxicity assay in which B16-F10 cells were co-cultured with activated T cells for 48 h and subject to CCK8, quantification using a spectrometer at OD (450 nm) followed by crystal violet staining (Fig. 5D). After co-cultured for 24 h, we observed that Ogr1-deficient in CD8^+^ T cells aggregated around the tumor cells, whereas WT cells were generally scattered and distributed (Supplementary Fig. 3C), also confirming that Ogr1 deficiency not only enhances the ability of T cells to move toward tumor cells but also caused them to kill tumor cells more efficiently (twofold increase) (Fig. 5D). Furthermore, Ogr1 deficiency undoubtedly resulted in increased TNF- α and granzyme B secretion by CD8^+^ T cells (Fig. 5E). To understand the functional consequences of melanoma acidosis induced by Ogr1 expression on T cells at the whole-genome transcription level, T cells were isolated from tumor-bearing mice and analyzed via RNA-sequencing. This analysis showed that genes representing T-cell activation and tumor-killing including *Ifng, Vcam1, Rgs1*, and *Il2rb*, had higher expression levels, whereas T-cell exhaustion genes such as *Tim-3, Ccl3, Rgs2*, and *Cd6f3*, had lower expression levels in *Ogr1*^*−/−*^ mice than that in the WT mice (Fig. 5F). Our in vivo immunoassay and RNA-Seq analysis demonstrated that *Ogr1*^*−/−*^ mice stimulated antitumor immune responses mainly composed of effector T cells, especially CD8^+^ T cells.

Collectively, these findings comprehensively indicate that Ogr1 inhibition reactivates T cells and has a cytotoxic role by reducing the activity of high glycolysis, resulting in comparatively low acidification of the TME and, subsequently aid in tumor suppression.

### Ogr1 may serve as a novel target for immunotherapy against cancer

**Fig. 6:**
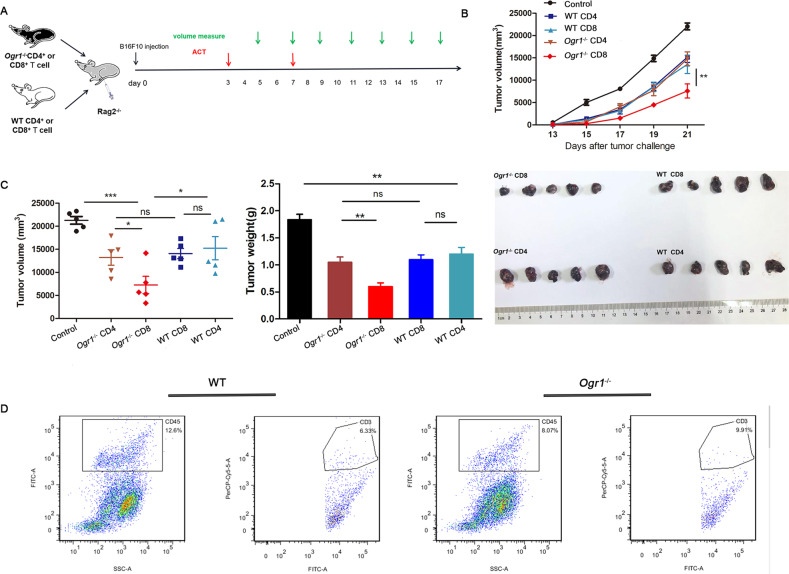
Ogr1 inhibition enhances the therapeutic response of ACT. **A** A schematic view of the treatment plan. In all, 2 × 10^5^ B16-F10 cells in a 100 µl volume were transplanted into 25 Rag2^−/−^ mice (*n* = 5). Seven days after transplantation, mice were randomized into five groups, and followed by treatments via intraperitoneal injection of PBS, *Ogr1*^*−/−*^ -CD4^+^/CD8^+^T cells or WT-CD4^+^/CD8^+^ T cells twice in the first week. Tumor growth was measured using calipers. **B**, **C** Summaries of tumor growth (**B**) and tumor volume (Day 21) (**C**) of Rag2^−/−^ (*n* = 6) after T-cell infusion. **D** Flow cytometry showed the proportion of CD45^+^ and CD3^+^ cells in tumor of mice treated with ACT. These experiments were repeated three times. The results are presented as the means ± SD. *P < 0.05, **P < 0.01, ***P < 0.001, as obtained by unpaired test.

ACT is a new type of treatment in which the antitumor activity of immune cells in patients with tumors is amplified in vitro and then transfused back to these patients [25]. There are also limitations to this treatment, such as the difficulty of immune cells entering the tumor parenchyma under normal conditions and their dysfunction when delivered back into the body [26]. Our previous experiments proved that *Ogr1*^*−/−*^ T cells had an enhanced ability to infiltrate tumor cores in an acidic environment. To address whether Ogr1 can serve as a target to improve the therapeutic effect of ACT on solid tumors, we created a mouse model, that could be used to study combination therapies. T cells (CD4^+^ T cells or CD8^+^ T cells) used as the source of adoptive transfer cells were stimulated with IL-2 in vitro after isolation from *Ogr1*^*−/−*^ and WT mice. When Rag2^−/−^ as recipient mice had formed palpable tumors, they were intravenously administered with stimulated T cells (1 × 10^7^) as a treatment regimen on the seventh and ninth days (Fig. 6A). To investigate whether T cells injected into Rag2^−/−^ mice tracked tumor cells, we killed two mice from each group to analyze the tumor tissues via flow cytometry. The presence of CD45^+^ cells was clearly observed in the grinding tumor tissue via flow cytometry, where the CD3^+^ cells found in tumors represented T cells (Fig. 6D). Treatment results showed that Rag2^−/−^ tumors were markedly reduced by infusing *Ogr1*^*−/−*^*-*CD8^+^ T cells, whereas the difference in tumor weight between the treatments of infused *Ogr1*^*−/−*^*-*CD4^+^ T cells and infused WT-CD8^+^ T cells did not significantly change (Fig. 6B, C). The above in vivo findings suggest a potential role of *Ogr1*^*−/−*^ CD8^+^ T cells in enhancing the therapeutic effect of ACT.

## Discussion

Tumor immunity is becoming a critical factor in the control and treatment of cancer. Spontaneous immune responses in cancer patients have been shown to modulate disease progression and positively influence prognosis [2]. Nevertheless, cancer immunotherapies such as CTLA-4 and PD-1 inhibitors are effective only in a minority of patients [27, 28], therefore, there is still an urgent need for strategies to expand the population that can benefit from these novel therapies [29, 30]. Recent studies have demonstrated that in addition to intracellular influences, one of the driving forces for the TME to become a hostile environment for antitumor immune cells comes from a series of biochemical reactions in which tumor cells induce local acidity by increasing glucose uptake and glycolysis while simultaneously stimulating an ATP-dependent proton extrusion mechanism [31]. Therefore, an acidic microenvironment represents a new framework for future research instructions in cancer research. However, to date, the molecular mechanisms of immune cell sensing and response to tumor acidosis have not been fully explored. The high concentration of tumor-derived lactic acid leads to the impairment of T-lymphocyte function, which can be achieved by increasing the apoptosis rate and reducing the number of cytokines [32–34]. To neutralize metabolic acid loading, immune cells use pH-sensing proteins to promote their survival in acidic environments [8]. Recent studies have shown that Ogr1, as a member of the proton-sensitive GPCR family, is overexpressed in T cells [10, 35], but the function of this receptor, especially in the presence of cancer-related acidity, remains elusive. In the present study, we systematically investigated the expression, effector function, and tumor-killing mechanism of Ogr1 on T-cell, and highlighted it as a potential target for cancer immunotherapy.

Previous studies have shown that Ogr1 expressed in tumors was initially described as a positive regulator of tumor initiation and progression and is associated with poor clinical prognosis [18, 36–38]. Recent studies have targeted Ogr1 in host immune cells. For example, Ogr1 promotes the differentiation of macrophages into the M2 type [18] and also paralyzes the migration of dendritic cells to the draining lymph nodes after exposure to antigens [39]. In our work, we indicated that the effect of Ogr1 on the host cells, rather than the tumor cells, was found to account for immune evasion. In melanoma models, the loss of Ogr1 in host cells significantly delayed tumor growth and metastasis as well as extended survival. Notably, Ogr1 knockdown in mice with B16-F10 tumors showed no significant change in tumor growth compared with WT mice. These data indicate that the host cell expression of Ogr1 has a dominant role in suppressing antitumor immunity. A high expression of Ogr1 in T cells has been reported at the cellular level [10]. Single-cell sequencing was carried out to reveal a key observation that Ogr1 deficiency can cause an increase in the number of tumor-infiltrating immune cells and a decrease in the number of tumor-promoting cells. Since in vivo testing revealed a remarkable increase in total CD8^+^ T-cell infiltrates in tumors of Ogr1^−/−^ mice, we further constructed mouse models of conditional knockout of Ogr1 in CD4^+^ and CD8^+^ T cells, which markedly resulted in the lightening of the tumor load.

A great deal of evidence shows that acidosis is one of the hallmarks of tumors and is also a key factor in tumor progression [40]. Melanoma cells typically exhibit high glycolytic activity, resulting in pH values as low as 6.0–6.7 in the TME due to lactic acid production [41–43]. The dorsal-window chambers are a microscope that can accurately examine the pH levels of a tumor and its microenvironment. By overlaying the acidity map created by Seminaphtolrhodafluor-1 vis IHC staining of LAMP2 in the tumor, the region where LAMP2 is overexpressed is matched with the acidic region [44]. We detected LAMP2 expression in isolated mouse tumors and found that host cell knockout of Ogr1 reduced LAMP2 expression in tumors, suggesting that the phenomenon of tumor acidosis was somewhat buffered. Furthermore, the high-serum expression of alternative markers of high glycolytic activity, such as LDH, MDH, and Pkm2, is associated with poor prognosis and is also negatively associated with overall survival with immunotherapy, suggesting that high glycolytic activity contributes to immune escape [21, 45–47]. It is worth noting that in the single-cell sequencing results, the factors involved in the high glycolytic activity in the Ogr1^−/−^ mice had a lower expression than those in the WT mice. We also found that in vitro pH to 6.6, the pH level most commonly detected in tumors resulted in tumor-specific CD8^+^ T lymphocytes being a non-responsive state [48, 49]. To simulate this process in vitro, we used hydrochloric acid to reduce the pH value in the cell medium to 6.6 [50], and tested the effect of Ogr1 on T cells in an acidic environment using T-cell function tests. In vitro, blocking Ogr1 at acidic pH reversed T-cell function by enhancing T-cell proliferation and migration. Moreover, the cytotoxicity assay also proved that blocking Ogr1 impelled T cells to migrate to the tumor’s boundary, and killed tumor cells by secreting sufficient granzyme B, TNF, and an appropriate amount of IFN-γ. The above phenomenon was not obvious in CD4^+^ T cells, possibly, owing to the reason may be the accumulation of M2-like tumor-associated macrophages, myeloid suppressor cells [51], and immunomodulatory DC cell types [52]. In addition, tumor cells in the TME can also interfere with the function of T cells by producing high levels of arginase and promoting the induction of Tregs [53, 54]. Although the total number of CD4^+^ T cells was increased in vitro, the number of Tregs did not change, resulting in a reduction in the antitumor effect in vivo. Based on these results, we will further improve the experiments performed. Combined with transcriptome sequencing, it was also demonstrated that Ogr1 deficiency promotes the rapid transformation of CD4^+^ and CD8^+^ T cells into effector/activated T cells. At the same time, it trigged the strongest signal in antitumor cytotoxic gene expression through upregulation of the pathways associated with cytokine–cytokine receptor interaction, antigen processing, and presentation, IL-17 signaling pathway, cytokine signaling pathway, among others. Remarkably, the secretion of TNF in *Ogr1*^*−/−*^ -T cells increased in vitro, which was consistent with the high expression of the TNF enrichment pathway obtained from the RNA-sequencing data. Furthermore, studies have shown that the chemotactic effects between CCL5, CCL9–10, CCL12, and T cells; CCL19, CCL21, and dendritic cells; CCL15 and B cells, which were found in the most enriched pathway associated with cytokine–cytokine receptor interaction, are ideal prospects applications tumor-specific immunotherapy [55, 56].

Targeting tumor acidity can inhibit tumor growth [57, 58], which further supports the idea that *Ogr1* is an attractive target for anticancer therapy development, perhaps as part of a combination therapy regimen. In the adoptive reinfusion experiment, T cells targeting Ogr1 showed excellent antitumor effects. However, until now, few compounds have been identified as selective Ogr1 ligands/modulators [59]. Based on yeast screening experiments for Ogr1, a new Ogr1 regulator was predicted based on 3.1 million molecules, and the benzodiazepine drug lorazepam was identified as a non-selective Ogr1-positive allosteric regulator. The same method has been used to screen GPR65 allosteric agonists and negative allosteric modulators, while the negative allosteric modulators of Ogr1 are yet to be identified. We believe that such compounds may be candidates as anticancer therapeutics, especially for tumors that rely on Ogr1 to enhance their growth or metastatic potential. Although PD-1, CTLA-4, LAG-3, and TIM-3 are equally important in CD8^+^ T cells, this may highlight the synergistic effect of Ogr1 and existing immunosuppressive checkpoint inhibitors in the treatment of advanced cancers.

## Conclusion

Our research is based on the phenomenon of high glycolytic activity in solid tumors, we found that the acidic microenvironment induces the expression of Ogr1 in T cells. Ogr1 inhibition reactivates T cells and has a cytotoxic role by reducing the activity of high glycolysis, resulting in comparatively low acidification of the TME and, subsequently aid in tumor suppression. In addition, adoptive transfer of Ogr1 deficiency in T cells enhanced the antitumor response, with the potential for immediate clinical transformation.

## Supplementary information

Supplementary Figure Legends

Supplementary Figure 1

Supplementary Figure 2

Supplementary Figure 3

## Data Availability

All data that support the findings of this study are available from the corresponding authors upon reasonable request.
