# Supplementary material for: Inhibition of host Ogr1 enhances effector CD8+ T-cell function by modulating acidic microenvironment
Source: Cancer Gene Ther. 2021 Jun 22;28(10-11):1213–24. doi: 10.1038/s41417-021-00354-0 (PMC8571096; doi:10.1038/s41417-021-00354-0)
Supplement: Supplementary file 1 — Supplementary Figure Legends [file 41417_2021_354_MOESM1_ESM.docx]

**Supplementary Figure 1.** **A** DNA agarose gel electrophoresis was used to detect and select that #1 was *Ogr1^-/-^* mice, #2-4 was Ogr1^WT/KO^ mice and #5 was Ogr1^WT/WT^ mice. **B** qRT-PCR was used to detect the expression of Ogr1 in different tissues of WT and KO mice. The PCR program was 94°C, 10 min; 40 cycles for GAPDH and Ogr1 (94°C, 20s; 60°C, 30s; ) both in tissues and T cells. **C** Lentivirus carrying shRNA to target Ogr1 was transfected to generate Ogr1-knockdown B16-F10 cell. **D** Ogr1-over-expressing lentivirus was transfected to generate Ogr1-high B16-F10 cells. **E** Tumor growth curves of subcutaneous B16-F10 or B16-F10-high*Ogr1* (5x10^5^ in 100 µl PBS) in WT (n=10) mice. **F** Weights and volumes of the melanomas and spleens collected on the 15th day. **G** Hematoxylin-eosin staining show the size of metastatic tumor in lung tissue between WT and *Ogr1^-/-^*.

**Supplementary Figure 2. A** The violin diagram of T cells displaying select marker gene expression. **B** The classification scale stack diagram of each cluster in *Ogr1^-/-^*-Tumor and WT-Tumor groups. **C, D** Enrichment analysis of Kyoto Encyclopedia of Genes and Genomes (KEGG) pathway showed top 10 pathways in *Ogr1^-/-^*(**C**) and WT group(**D**).

**Supplementary Figure 3. A** After staining with anti-CD3, anti-CD4 or anti-CD8a, cellar purity was determined by flow FACS, which was 91% and 87%, respectively. **B** Representative figures displaying proliferation of *Ogr1^-/-^* CD8^+^ T cells after anti-CD3 and anti-CD28 antibody stimulation at pH7.2 or pH6.4 under the fluorescence microscopen. **C** Representative images show that B16-F10 cells were co-cultured with activated CD8+T cells at 4 h and 24 h.
