# Supplementary figures and images for: Inhibition of host Ogr1 enhances effector CD8+ T-cell function by modulating acidic microenvironment
Source: Cancer Gene Ther. 2021 Jun 22;28(10-11):1213–24. doi: 10.1038/s41417-021-00354-0 (PMC8571096; doi:10.1038/s41417-021-00354-0)

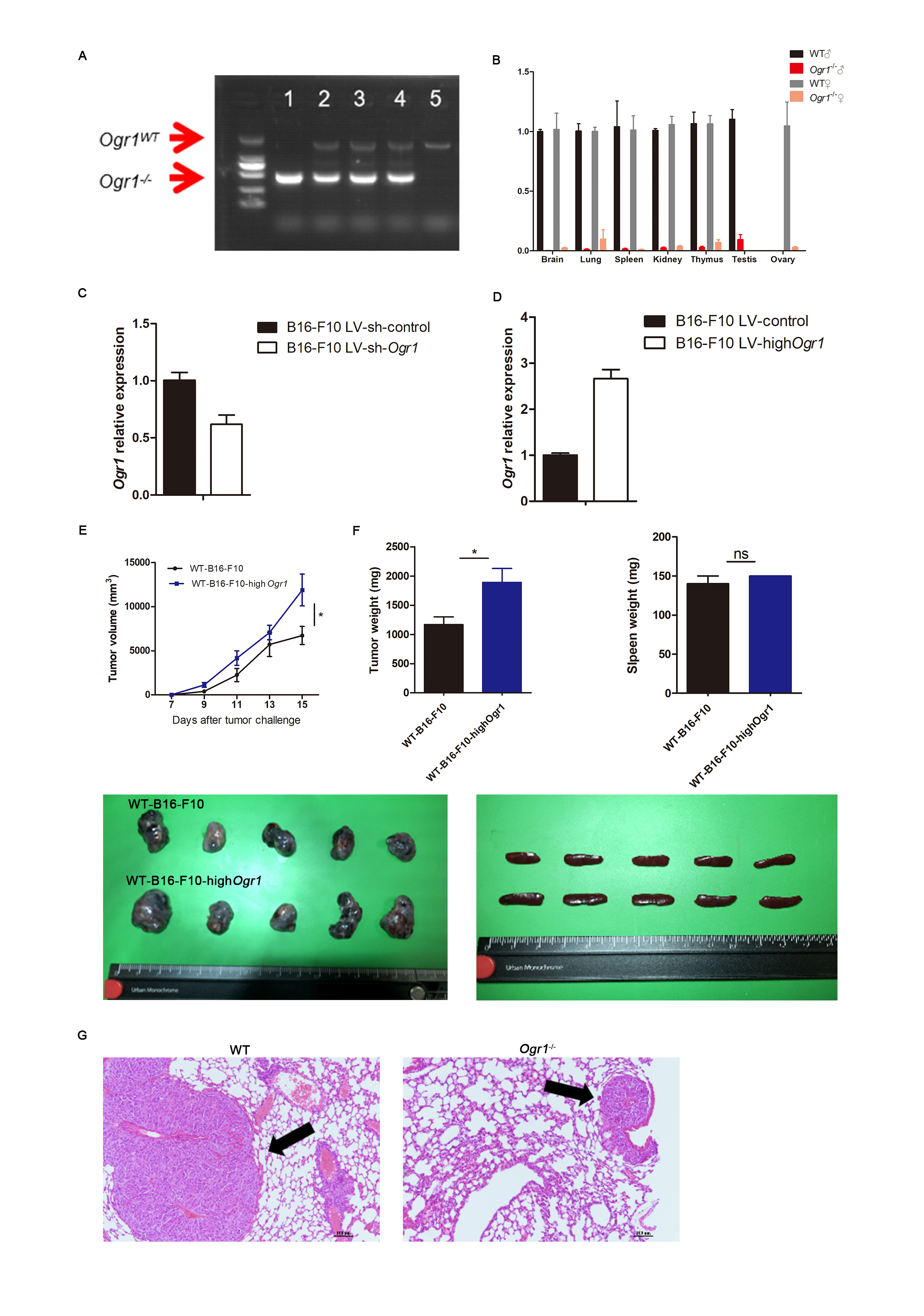

Supplement: Supplementary file 2 — Supplementary Figure 1 [file 41417_2021_354_MOESM2_ESM.png]

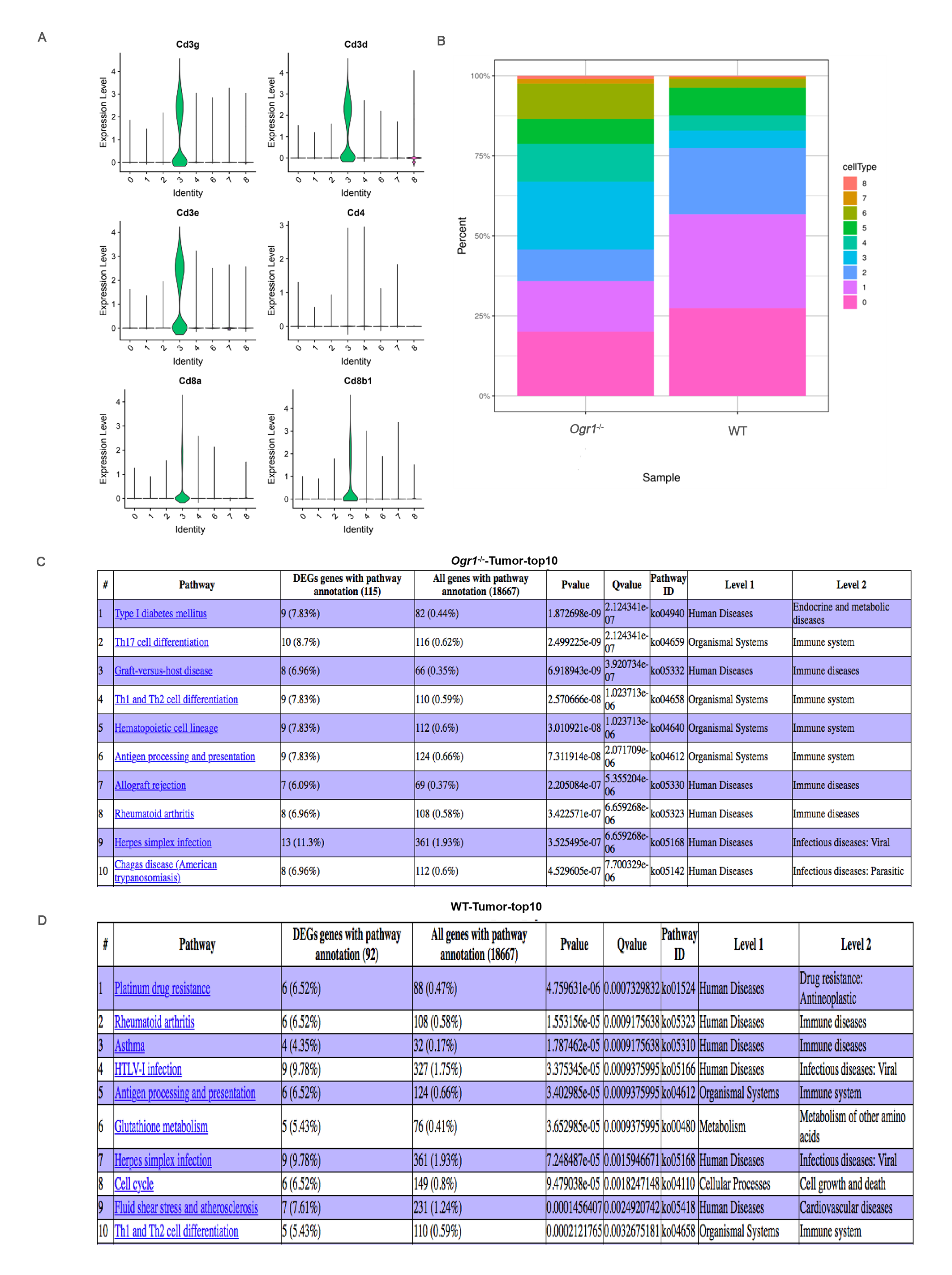

Supplement: Supplementary file 3 — Supplementary Figure 2 [file 41417_2021_354_MOESM3_ESM.png]

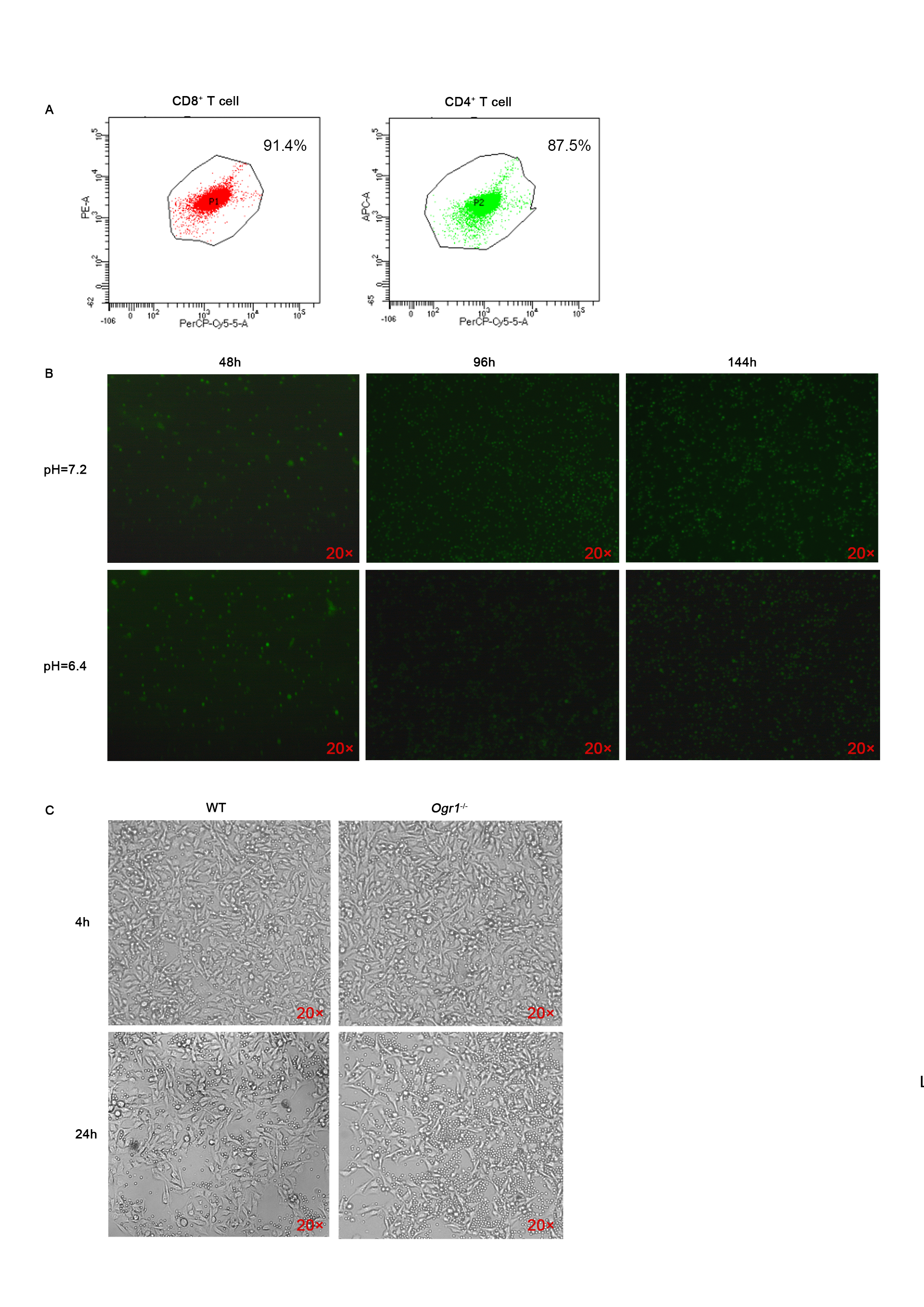

Supplement: Supplementary file 4 — Supplementary Figure 3 [file 41417_2021_354_MOESM4_ESM.png]
